# Queen Victoria

**Published:** 1922-03

**Authors:** 


### THE REVIEW OF THE MONTH.

### QUEEN VICTORIA.

If we place the " Life of Queen Victoria," by Mr. Lytton Strachey, as we cannot fail to do, side by side with his earlier volume of four biographies entitled " Eminent Victorians," a very marked resemblance will appear. Both books are part of the same scheme. While our late gracious Queen appears to us in general as a modern Gloriana, the centre of a group of remarkable men such as the world's history presents only during occasional long periods of widespread prosperity, to Mr. Lytton's eyes her personal influence does not account either for the great events that happened in her time or for the eminence of the chief characters which surrounded her. We see in his biography, what is lacking in the earlier book, a sincere attempt to refrain from belittling his subject, but certainly no endeavour to place her on a pinnacle of exceptional fame. It is, in fact, an echo of Mr. Dooley's claim to be of equal eminence with Queen Victoria, since the great steps of world progress which happened in her time occurred also in his own.

In spite of obvious temptations, the writer does not much indulge in the irony of which he is a master, and he makes none at the expense of Royalty itself. Even Prince Albert, for long a mark for shafts of British wit, escapes sarcasm, with the single exception of his efforts in improving the level of art prevailing in his adopted country. Albert, indeed, stands out as the central figure in the book whose future influence is implicit in the whole history of Victoria's upbringing and whose memory coloured the whole period of her widowhood during a period when she seriously endeavoured to assert her personal influence as monarch. In a very remarkable passage commenting on the Prince's death, with which the writer does not agree, Mr. Strachey supposes that if his life had been prolonged, " an attempt might have been made to convert England into a state as exactly organised, as elaborately trained, as efficiently equipped and as automatically controlled, as Prussia herself." In spite of Disraeli's authority for this anticipation, it is probable that the result of such an attempt, which no one but a Royal biographer would be tempted to suggest as possible, would have been a Radical, if not a Chartist, revolution during the '80's, or perhaps even in the '70's. Albert's constitutional discretion would never have invited such a catastrophe.

If the present volume be closely analysed it will appear as a group of male biographies, and those not of the greatest importance in the country. Where are the women ? The Duchess of Kent is a shadow quickly disposed of. Even the grim Baroness Lehzen, who probably had more effect in moulding Victoria's character than any other in youth, melts like a wax figure before the rising sun of Albert of Coburg. Victoria's own identity is a reflection of only three or four men, whom she trusted implicitly ; of Melbourne, when she was feeling her way towards filling her great hereditary position ; of Albert, through her concentrated love for him, her admiration of his splendid person, character and accomplishments, and her deep respect for his industry and self-sacrifice to duty ; of Disraeli, because she succumbed to the deftness of his flattery, the genuineness of his devotion to the Queen and woman, and the exceptional capacity with which he managed all with whom he came in contact. After Disraeli's fall from power she gave herself up unreservedly to her inner life and her silent companionship in spirit with John Brown, who remains rather a ludicrous figure on the Royal platform.

Only on one occasion, the Russian crisis of 1878, faithful to her interpretation of Albert's European policy, did she make any violent attempt to coerce her favourite minister in a matter of first-class policy, when, according to the record of her own letters, she appears to have approached the point of madness in her endeavour to compel her ministers and to force the country into war. Yet in the end she gave evidence of that remarkable quality of discretion in which she had been trained by her husband. On this and on similar occasions her vehemence was checked by her ingrained experience and her native shrewdness. " She might," says Mr. Strachey, " oppose her Ministers with extraordinary violence ; she might remain utterly impervious to arguments and supplications ; the pertinacity of her resolution might seem unconquerable ; but at the very last moment of all, her obstinacy would give way. Her innate respect and capacity for business, and perhaps, too, the memory of Albert's scrupulous avoidance of extreme courses, prevented her from ever entering an *impasse*. By instinct she understood when the facts were too much for her, and to them she invariably yielded."

Of other men intimately delineated in the circle of the Queen, or just beyond, there are admirable portraits of King Leopold, Baron Stockmar and Lord Palmerston. The latter was essentially different from his Royal superiors—" He lived by instinct, by a quick eye and a strong hand, a dexterous management of every crisis as it arose, a half-unconscious sense of the vital elements in a situation. He was very bold . . . but there is a point beyond which boldness becomes rashness, a point perceptible only to intuition and not to reason ; and beyond that point Palmerston never went." Other Premiers are here very scantily treated. Sir Robert Peel and Gladstone are mentioned only for their deficiencies. Lords Aberdeen, Derby and Rosebery are given the go-by.

Of medical history there is only one case treated, the unfortunate complication of Sir James Clarke and Lady Flora Hastings when the physician, owing possibly to too great a deference to Court opinion, made a deplorable mistake. It was an incident only worth mention because it led to an undue reliance by the Queen on a physician whose treatment of Prince Albert's last illness has met with severe criticism. In the medical Victorian chronicles our readers may be recommended to turn to Sir Squire Sprigge's " Physic and Fiction," reviewed elsewhere in our columns.—G. B. D.

